# Comprehensiveness, readability, and reliability of Brazilian websites available for lay people’s guidance on adenotonsillectomy

**DOI:** 10.1016/j.bjorl.2019.06.012

**Published:** 2019-07-27

**Authors:** Sofia Prata Piña, Camila de Castro Corrêa, Lídia Raquel de Carvalho, Silke Anna Theresa Weber

**Affiliations:** a Universidade Estadual Paulista (UNESP), Faculdade de Medicina de Botucatu, Departamento de Oftalmologia, Otorrinolaringologia e Cirurgia de Cabeça e Pescoço, Botucatu, São Paulo, Brazil; b Universidade Estadual Paulista (UNESP), Faculdade de Medicina de Botucatu, Departamento de Bioestatística, Botucatu, São Paulo, Brazil

**Keywords:** Internet, Tonsillectomy, Ethics, Communication barriers

## Abstract

**Introduction:**

Adenotonsillectomy is an invasive surgical procedure, which may encourage the search for information by the lay population through online search engines. The quality of this information, however, requires an evaluation due to the wide diversity of the available content.

**Objective:**

To evaluate the quality, in terms of ethical principles, readability and comprehensiveness, of the most accessed websites concerning guidance to parents/guardians related to their children’s recommended adenotonsillectomy.

**Methods:**

The websites mentioned on the first 10 pages obtained after the search using “removal”, “tonsils” and “adenoids” as keywords, after applying the inclusion and exclusion criteria, were selected. All were assessed using the Flesch Readability Index and Health on the Net Code tools, in addition to an evaluation of the content by two independent evaluators. The data were described, and the inter-rater agreement was calculated by the Kappa coefficient.

**Results:**

34 websites were found, of the 100 assessed ones, which met the inclusion and exclusion criteria using the Google and Yahoo! Tools. Sixteen (47%) pages were considered reasonably difficult/difficult to read according to the Flesch Readability Index. Most of them met less than half of the analyzed ethical requirements according to the Health on the Net Code, and the overall comprehensiveness average was considered insufficient.

**Conclusion:**

A deficit of overall quality (comprehensiveness, readability, and ethical principles) was demonstrated for the websites available to parents or guardians about the adenotonsillectomy procedure in children.

## Introduction

Adenotonsillectomy is the surgical procedure carried out to remove the palatine and pharyngeal tonsils, being most frequently performed in the pediatric population. It is indicated in cases of hypertrophy of these tonsils accompanied by obstruction and recurrent infections in the upper respiratory tract, and may be associated with other conditions, such as otitis media and obstructive sleep apnea.^1^

Because it is an invasive procedure which requires general anesthesia, some parents of children scheduled to undergo the surgery tend to feel insecure. The search for more information about the procedure itself, with pre- and postoperative recommendations, along with the possible risks associated with surgery, is common among parents and families. It is observed that this search occurs mainly through the internet, specifically through the search engines Google and Yahoo!, and that this information obtained through the Internet has frequently influenced the parents’ decisions about the procedure proposed for their children.^2^

Given the influence of information obtained virtually on the attitudes of parents/guardians, an important aspect to consider is the information’s level of readability. The websites about the pre- and postoperative recommendations for tonsillectomy have shown a low readability, making it difficult to understand and interpret the information. Often these sites provide contradictory and outdated information, creating difficulty in making informed decisions regarding the care of their children.^3^, ^4^

Very often, the websites that are better evaluated by Internet users and the most often accessed ones do not always present the most up-to-date information. Thus, it is imperative to emphasize the importance of health professionals in providing reliable and evidence-based sources of information to patients, aiming to prevent the dissemination of incorrect/outdated information.^4^, ^5^, ^6^

These literature findings report the difficulty in obtaining reliable data on adenotonsillectomy. There have been no evaluations about the quality and readability of Brazilian websites, which encouraged us to perform this study.

## Objective

To evaluate the quality, in terms of ethical principles, readability, and comprehensiveness of the most often accessed websites containing guidance to parents/guardians of children with an indication for adenotonsillectomy.

## Methods

For the proposed investigation, the study was designed in two stages: search of the websites and evaluation of the websites.

### 1st stage: search of the websites

Google and Yahoo! search tools were used with the keywords “removal”, “tonsils” and “adenoids”, during the first semester of 2018. The keywords were chosen after an initial search using words that included, in addition to the ones chosen, the terms “adenotonsillectomy” and “tonsillectomy”. However, these last two words obtained results with a predominance of scientific articles that are not aimed at the lay population, justifying the non-use of these words for the purpose of this study.

The search included the first 10 pages of the two search engines with the pre-established descriptors, that is, 100 websites for each search engine. The inclusion criterion established that websites specialized in otorhinolaryngology would be selected, since this profession is responsible for performing the surgery. Websites that did not intend to clarify doubts, either on the procedure or on adenotonsillar hypertrophy, the main indication for surgery, were excluded. Thus, we excluded sources whose purpose was to report facts or share personal experiences, scientific articles, YouTube videos, news websites and online group discussion forums.

The website inclusion was performed by two judges, who selected the websites that were subsequently evaluated, considering the pre-established criteria. In cases of disagreement, these were reviewed by a third judge, so that a consensus could be established.

### 2nd stage: evaluation of websites

The websites were analyzed according to two protocols established in the literature: the Flesch Reading Test, which investigates the level of readability, and Health on the Net Code (HONCode), which scores the ethical principles. In addition to these, a qualitative assessment of the content coverage was performed. Only the specific pages were considered for all the analyses, without including the related links mentioned on the pages.

The Flesch Reading Test calculates the readability level automatically, considering the average length of the sentences and the average number of syllables per word.^7^ Therefore, the entire content was copied to Microsoft Word, part of the Office 2010 package, and at the end of the grammar review, the Flesch Reading Ease Index was given as a percentage, from 0 to 100, where 0%–30% is classified as Very difficult (technical terms), 30%–50% is Difficult (requiring High School or College/University level of schooling), 50%–60% is Reasonably difficult, 60%–70% is Standard (requiring an Elementary level of schooling), 70%–80% is considered reasonably easy, 80%–90% is Easy and 90%–100% is considered Very easy (requiring Kindergarten/Elementary level of schooling).^8^

The modified Health on the Net Code (HONCode) protocol was used to analyze ethics, to score property issues, purpose classification, authorship, author qualification, attribution, interactivity and updating. All of these items were scored as 0, 1 or 2, except for the first property item that was scored as 0 or 1. The higher the score (0–13), the better the website quality.^9^, ^10^ Thus, the analysis considered whether the website met more or less than 50% of the ethical principles, and which were the most and the least frequently considered issues in general.

Finally, the comprehensiveness of each website was assessed, in which the judges observed whether or not the contents included the following themes, attributing 1 (very insufficient) to 5 (very satisfactory) points for each question, based on an answer pattern previously created by the judges, supported by the knowledge acquired in medical practice and in the scientific literature,^11^ which allowed the scoring of the most relevant aspects for the guidance of patients in this situation, as listed below:

Definition of adenotonsillectomy

It is a surgical procedure performed under general anesthesia to remove the palatine/pharyngeal tonsils;

The surgery is performed through the mouth; there is no incision in the skin;

Sutures / stitches are not always necessary, and do not need to be removed after they surgery.

When is the surgery indicated?

Hypertrophy of the palatine/pharyngeal tonsils that cause daytime / nocturnal breathing difficulties;

Frequent throat infections;

After a peritonsillar abscess or recurrent complaints of caseous tonsillitis.

Risks

Risks of general anesthesia;

Bleeding during surgery and in the postoperative period;

Soon after the surgery the following can occur: fever, vomiting that can cause dehydration, sore throat/ear pain.

Benefits

Improved breathing, sleeping and quality of life.

Preoperative recommendations

Fasting

Postoperative recommendations

Cold liquids and foods;

Normal diet one week after the procedure;

One week of rest without physical activities or sun exposure.

A comprehensiveness score was obtained for each website based on the average of each item. The results were tabulated and submitted to descriptive analysis (mean and standard deviation). For the evaluation of the judges’ agreement regarding the HONCode and the comprehensiveness, the Kappa coefficient analysis was applied, indicating the amount of divergence in the inter-rater interpretation. Thus, agreement was classified as: No agreement, for Kappa value = 0; Minimum agreement = 0.01–0.20; Reasonable agreement = 0.21–0.40; Moderate agreement = 0.41–0.60; Substantial agreement = 0.61–0.80 and Perfect agreement = 0.81–1.00.^12^

## Results

The websites that met the inclusion and exclusion criteria were selected. In the Google search, 28 websites were selected of the 100 possible ones, while of the 100 websites found at Yahoo!, 33 were considered, of which only 8 were different from those obtained first in Google. Two pages were excluded during the analysis because they had been “taken down”. Therefore, 34 websites were included, constituting the final analyzed list, as detailed below (following the location order):

W01 ‒ http://portalotorrino.com.br/cirurgias/cirurgia-amigdalas-e-adenoides/

W02 ‒ http://kurahashimelo.com.br/Cuidados-Pre-e-Pos-Operatorio/cuidados-pos-operatorios-para-cirurgia-das-amigadas-ou-amigdalas-adenoide.html

W03 ‒ http://www.jamal.com.br/cirurgia-das-amigdalas-e-adenoides/

W04 ‒ http://www.clinicaorl.com/pos-op-amigdalas-e-adenoides/

W05 ‒ https://www.otorrino.pro/content/orientacoes-pos-operatorias

W06 ‒ http://www.inof.com.br/profissionais/cirurgia-das-amigdalas-eou-adenoides/

W07 ‒ http://www.drbrunobarros.com.br/pos-operatorio-adenoamigdalectomia

W08 ‒ http://www.cemahospital.com.br/adenoamigdalectomia/

W09 ‒ http://www.clinicaotorrinocenter.med.br/otorrino/cirurgia-das-amigdalas-e-adenoides/

W10 ‒ http://portalotorrino.site.med.br/index.asp?PageName=Cirurgia-20das-20Am-EDgdalas-20e-20Aden-F3ides

W11 ‒ http://www.otorrino-lavinsky.com.br/amigdalas-e-adenoides/

W12 ‒ https://www.ipo.med.br/pos-operatorio

W13 ‒ http://www.otovita.com.br/amigdalectomia-e-adenoidectomia.html

W14 ‒ http://www.leonardosa.com.br/website/index.php?option=com_content&view=article&id=105:passo-a-passo-na-cirurgia-de-garganta-07-cuidados-no-pos-operatorio&catid=62:doencas&Itemid=96

W15 ‒ http://www.otoalpha.com.br/tratamentos/hipertrofia-amigdalas-adenoides.html

W16 ‒ http://www.drlucianorotella.com/pos-operatorio/

W17 ‒ http://www.claudiaciuffi.com.br/hipertrofia-das-amidalas-e-adenoides/

W18 ‒ http://otorrinoclinicabh.com.br/patologias/amigdalas-e-adenoide/

W19 ‒ http://www.otorrinocruzvermelha.com/adenoamigdalectomia.php

W20 ‒ http://otorrinopediatria.tripod.com/poamigdalas.htm

W21 ‒ http://www.paulosaraceniotorrino.com.br/amigdalas-e-adenoides/

W22 ‒ http://www.fabriciopandini.com.br/procedimentos/cirurgia-das-amigdalas-e-adenoide/

W23 ‒ http://www.drmarioesposito.com.br/procedimentos/adenoamigdalectomia-adenoide-e-amigdalas

W24 ‒ http://www.hospitaldeotorrino.com.br/instrucao/pos-operatoria

W25 ‒ http://otorrinoscuritiba.com.br/saude/saiba-como-identificar-problemas-nas-amigdalas-e-adenoide.html

W26 ‒ https://drmurilolima.com/tag/cirurgia-adenoide-e-amidalas-preco/

W27 ‒ http://forl.org.br/InformacaoDoenca/Visualizar/19

W28 ‒ http://www.clinicadrcastagno.com.br/Pagina/40/Cirurgias#ADENOIDECTIOMIA

W29 ‒ http://cliaod.com/2018/04/10/quais-as-7-coisas-que-eu-preciso-saber-sobre-adenoides-e-amigdalas/

W30 ‒ http://www.clinicastefanini.com.br/hipertrofia-das-amigdalas-e-adenoides/

W31 ‒ http://www.otorrinospoa.com.br/index.php/rinossinusologia/35-adenoide-e-amigdalas

W32 ‒ http://www.noasp.com.br/artigos/otorrinopediatria/pos-operatorio-amigdalas-adenoide.php

W33 ‒ http://otorrinoclinicabh.com.br/patologias/amigdalas-e-adenoide/

W34 ‒ http://faccialece.com.br/hipertrofia-das-amigdalas-e-adenoide/

Some of the websites used an academic/scientific approach regarding Waldeyer's lymphatic ring structures and associated pathologies, not providing information on the adenotonsillectomy procedure for parents/guardians of patients with a surgical indication.

Regarding readability, according to the Flesch index, almost half (47%) of the websites were considered reasonably difficult/difficult (Table 1), requiring the equivalent of High School/College/University level of schooling. When analyzing the first 10 located websites, only one showed a standard level for reading.

**Table 1:** Results of the percentage of readability level according to the Flesch Readability Index.

	Score (%)	Classification		Score (%)	Classification
**W01**	47	Difficult	**W18**	50	Reasonably difficult
**W02**	45	Difficult	**W19**	42	Difficult
**W03**	58	Reasonably difficult	**W20**	53	Reasonably difficult
**W04**	65	Standard	**W21**	35	Difficult
**W05**	38	Difficult	**W22**	66	Standard
**W06**	53	Reasonably difficult	**W23**	49	Difficult
**W07**	54	Reasonably difficult	**W24**	51	Reasonably difficult
**W08**	36	Difficult	**W25**	44	Difficult
**W09**	47	Difficult	**W26**	44	Difficult
**W10**	54	Reasonably difficult	**W27**	60	Standard
**W11**	42	Difficult	**W28**	49	Difficult
**W12**	66	Standard	**W29**	51	Reasonably difficult
**W13**	34	Difficult	**W30**	34	Difficult
**W14**	57	Reasonably difficult	**W31**	32	Difficult
**W15**	40	Difficult	**W32**	52	Reasonably difficult
**W16**	55	Reasonably difficult	**W33**	50	Reasonably difficult
**W17**	49	Difficult	**W34**	25	Very difficult

As for the ethical issues, the mean score of the websites was 5.47. In 11 accessed pages, the author's qualification was only present in other tabs/links or showed more details on other pages of the website. The Property and Interactivity topics were the most frequently respected ones, while Attributions showed the greatest deficits. Only 9 pages (26.47%) met more than half of the ethical principles (Table 2).

**Table 2:** Average scores of the judges on ethical principles based on the modified Health on Net Code and the value and classification of agreement by the judges using the Kappa coefficient.

	Property	Purpose	Authorship	Author’s qualification	Attribution	Interactivity	Update	Overall score ± SD
**HON code**								
W01	1	1	2	2	0	1.5	0	7.50 ± 0.84
W02	1	1	0	0.5	0	2	0	4.50 ± 0.75
W03	1	1	2	2	0	2	1.5	9.50 ± 0.75
W04	1	1	0.5	0.5	0	2	0	5.00 ± 0.70
W05	1	1	1	0.5	0	2	1.5	7.00 ± 0.65
W06	1	1	0	0	0	2	0.5	4.50 ± 0.75
W07	0.5	1	1	1	0	2	0	5.50 ± 0.70
W08	1	1	0	0	0	1.5	0	3.50 ± 0.65
W09	1	1	0	0	1.5	2	1.5	7.00 ± 0.76
W10	1	0.5	1	1	0	0	0	3.50 ± 0.50
W11	1	1	0	0.5	0	1	0	3.50 ± 0.50
W12	0.5	1	0	0	0	2	0	3.50 ± 0.76
W13	1	1	0	0	0	2	0	4.00 ± 0.79
W14	0.5	1	1	1	0	2	0.5	6.00 ± 0.63
W15	1	1	0	0	0	2	0.5	4.50 ± 0.75
W16	1	1	1	1	0	1	0.5	5.50 ± 0.39
W17	1	1	1	1	0	2	0	6.00 ± 0.69
W18	1	1	0	0.5	0	2	0	4.50 ± 0.75
W19	1	1	0	0	1	1	0	4.00 ± 0.53
W20	0.5	1	2	2	0	2	0	7.50 ± 0.93
W21	1	1	1	1	1.5	2	1.5	9.00 ± 0.39
W22	1	1	0.5	1	0	2	0	5.50 ± 0.70
W23	0.5	1	0.5	1	0	2	0.5	5.50 ± 0.64
W24	1	1	0	0	0	2	0.5	4.50 ± 0.75
W25	0.5	1	2	2	0	2	1.5	9.00 ± 0.81
W26	1	1	1.5	1.5	0	1	0.5	6.50 ± 0.53
W27	1	1	0	0	0	2	0	4.00 ± 0.79
W28	1	1	0	0.5	0	2	0	4.50 ± 0.75
W29	1	1	0	1	1	2	1.5	7.50 ± 0.61
W30	0.5	1	0.5	0.5	0	2	0.5	5.00 ± 0.64
W31	0.5	1	0	0	0	1	0	2.50 ± 0.48
W32	1	1	0	0	0	2	1.5	5.50 ± 0.81
W33	1	1	0.5	1	0	2	0	5.50 ± 0.70
W34	1	1	0	0.5	0	2	0.5	5.00 ± 0.70
Mean	0.88 ± 0.22	0.99 ± 0.09	0.56 ± 0.69	0.69 ± 0.65	0.15 ± 0.42	1.76 ± 0.48	0.44 ± 0.59	5.47 ± 0.51
**Kappa**								
Value (CI)	0.41 (0.11; 0.70)	0.87 (0.63; 0.99)	0.77 (0.59; 0.95)	0.61 (0.38; 0.83)	0.25 (0.01; 0.55)	0.84 (0.65; 0.99)	0.27 (0.08; 0.45)	
Classification	Moderate agreement	Perfect agreement	Substantial agreement	Substantial agreement	Reasonable agreement	Perfect agreement	Reasonable agreement	

CI, confidence interval.

The Kappa Agreement Coefficient showed Substantial or Perfect agreement for Purpose, Authorship, Author's Qualification and Interactivity at the HONCode analysis. The rating was Reasonable for Attributions and Updates. None of the topics obtained “no agreement” or “minimum agreement” (<0.20) classification, and the others showed moderate agreement (Table 2).

Regarding the item comprehensiveness, the mean of the websites was 2.33, corresponding to an "Insufficient" comprehensiveness according to the adopted criteria. The classification was “Regular” for 6 pages (17.64%), and the remaining 28 (82.36%) pages were classified as “Insufficient” or “Very Insufficient”. The “Indications” item was most frequently contemplated by the websites, obtaining the overall mean of 3.1 (Regular). The topics “Preoperative Benefits” and “Recommendations” were the most deficient ones in the websites, obtaining means of 1.25 and 1.69 (Very Insufficient), respectively. The “Satisfactory” or “Very Satisfactory” classification was observed at a higher frequency for the topic of “Postoperative Recommendations”, with 17 pages showing such classification (Table 3).

**Table 3:** Results on average of the judges’ evaluation of the comprehensiveness (on definition, indication, risks, benefits, pre- and post-surgical recommendations) and the value and classification of agreement of the judges using the Kappa coefficient.

	Definition	Indication	Risks	Benefits	Pre-surgical recommendations	Post-surgical recommendations	Mean ± sd	Classification
**Comprehensiveness**								
W01	2.5	4.5	2.5	1	1	5	2.75 ± 1.70	Insufficient
W02	1	1	3.5	1	1	5	2.08 ± 1.74	Insufficient
W03	1.5	5	4	1.5	1	3	2.67 ± 1.60	Insufficient
W04	1	1	2	1	1	4	1.67 ± 1.21	Very insufficient
W05	1	1	4	1	1	5	2.17 ± 1.83	Insufficient
W06	2.5	1	4.5	1	5	4.5	3.08 ± 1.83	Regular
W07	4	2.5	3	1	1	4	2.58 ± 1.36	Insufficient
W08	4	5	1	1	2	4	2.83 ± 1.72	Insufficient
W09	2.5	4.5	2.5	1	1	5	2.75 ± 1.70	Insufficient
W10	2.5	3.5	2.5	3	4.5	4.5	3.42 ± 0.92	Regular
W11	5	4.5	4	1	4	1.5	3.33 ± 1.66	Regular
W12	1	1	2.5	1	1	4.5	1.83 ± 1.44	Very insufficient
W13	3.5	5	1	2	5	1	2.92 ± 1.86	Insufficient
W14	1	1	1	1	1	4.5	1.58 ± 1.43	Very insufficient
W15	2.5	4	1	2	1	1	1.92 ± 1.20	Very insufficient
W16	1	1	2.5	1	1	4	1.75 ± 1.25	Very insufficient
W17	5	4	1.5	2.5	1	3	2.83 ± 1.51	Insufficient
W18	2.5	3	1	1	1	1	1.58 ± 0.92	Very insufficient
W19	4	5	1.5	1.5	1	1	2.33 ± 1.72	Insufficient
W20	4	2	4	1	1	4	2.67 ± 1.51	Insufficient
W21	3	3.5	1	1	1	1	1.75 ± 1.17	Very insufficient
W22	3.5	2	1.5	3	1	1.5	2.08 ± 0.97	Insufficient
W23	3.5	5	4.5	1	1	3.5	3.08 ± 1.72	Regular
W24	1	1	3.5	1	1	4.5	2.00 ± 1.58	Insufficient
W25	3	2.5	1	1	1	1.5	1.67 ± 0.88	Very insufficient
W26	2.5	4	3	1	2	1	2.25 ± 1.17	Insufficient
W27	2.5	4.5	2.5	1	4	4	3.08 ± 1.32	Regular
W28	4.5	3.5	4.5	1	5	4	3.75 ± 1.44	Regular
W29	2	3	1	1	1	1	1.50 ± 0.84	Very insufficient
W30	2.5	3	1.5	1	1	1	1.67 ± 0.88	Very insufficient
W31	2.5	4.5	1	1	1	1	1.83 ± 1.44	Very insufficient
W32	4	3.5	4	1	1	4	2.92 ± 1.50	Insufficient
W33	1.5	3	1	1	1	1	1.42 ± 0.80	Very insufficient
W34	1.5	3	1	1	1	1	1.42 ± 0.80	Very insufficient
Mean	2.63 ± 1.23	3.1 ± 1.45	2.37 ± 1.28	1.25 ± 0.57	1.69 ± 1.39	2.93 ± 1.61	2.33 ± 0.72	Insufficient
**Kappa**								
Value (CI)	0.34 (0.17; 0.50)	0.69 (0.54; 0.84)	0.53 (0.34; 0.72)	0.22 (0.01; 0.50)	0.58 (0.34; 0.83)	0.83 (0.73; 0.92)		
Classification	Reasonable agreement	Substantial agreement	Moderate agreement	Reasonable agreement	Moderate agreement	Perfect agreement		

CI, confidence interval.

Regarding comprehensiveness, the Coefficient showed Good or Very Good agreement for “Indications” and “Postoperative period” in the comprehensiveness analysis. The classification was “Regular” for Definition and Benefits and none of the topics obtained a “Poor” classification (<0.20). The other topics showed “Moderate agreement” (Table 3).

## Discussion

In the increasingly digitized world, one needs to be concerned about the information available for our patients. To evaluate the quality of these websites, several aspects must be considered, not just their content. One must be aware that even websites with adequate content quality may not convey the desired information due to the presence of possible communication barriers and the influence of users’ individual processing. The Brazilian population has easy access to the internet and its content but has difficulty interpreting texts. 50.99% of Brazilian students are below the basic reading proficiency level according to PISA — International Program of Student Assessment of 2015,^13^ justifying the evaluation of the content and readability of websites available to the lay population carried out the present study.

In our study, the first 10 pages of each search engine were included, surpassing the average number of pages commonly accessed in these search engines, which is usually around 2–3 pages.^13^, ^14^ This resulted in a larger sample with 34 websites included, while previous studies selected 14,^15^ 15^16^ and 30^3^ websites aimed at the providing guidance to parents/guardians of children with adenotonsillectomy indications.

The presence of websites related to scientific and/or academic information about Waldeyer's lymphatic ring structures was observed, which did not aim at providing explanations about adenotonsillectomy, highlighting the difficulty for the lay population to find websites with the desired information profile.

Knowing that 74% of the people state that they seek medical information on the internet, and that only half have full College/University education,^17^ it is noteworthy how harmful it is to use a language that is scarcely accessible in the media aiming to disseminate knowledge of medical importance. In this study, the readability assessment showed to be “difficult” for more than half of the websites (Table 1), which may interfere with the understanding and assimilation of the content, especially for users with lower educational levels. Another study,^16^ using the same Flesch Reading Index tool, observed a score of 42.3 points, similar to ours of 47.8, warning that there are flaws related to inadequate language use not only in Brazil.

When analyzing ethical principles, only 9 (26.47%) pages met more than 50% of the items evaluated by HONCode. In 32.35% of them, information such as the author's qualification was not shown on the first page, requiring the user to access other pages to obtain this information. Similarly, additional information on the subject was found only in other links on the website, making it easier to deviate from the focus of the original question, in addition to making the search more tiresome for the user.

The topics “Property” and “Interactivity” were the most often respected ones, frequently highlighting the page developer and phone/contact links, respectively. “Attributions” showed the biggest deficits, as many of the websites did not make it clear the source of information provided by the page, reducing the reliability of the available content.

Other studies used the DISCERN tool to evaluate aspects similar to those observed by HONCode. Of 80 points, the average score obtained was 55.1 for websites in English^16^ and 37.6 for pages in Turkish.^15^ Comparatively, the score of the Brazilian websites obtained 5.5 out of 13 possible points, closer to the Turkish reality, with greater deficit in relation to the websites in the English language.

A lack of quality was observed, based on the adopted criteria, regarding the provided information. More than half of the websites were considered insufficient regarding the extent of knowledge about adenotonsillectomy (Table 3). Relevant topics, such as preoperative recommendations, may not be adequately clarified, since 76.47% of the websites had information considered to be very insufficient in this area. Thus, the parents/guardians of children scheduled to undergo adenotonsillectomy do not reach the purpose of the search aiming to complement their knowledge through the internet, to encourage their decisions about this surgical procedure.

When evaluating the three best websites for readability, ethical principles and comprehensiveness, it was observed that different websites were classified for each category. This demonstrates the variability of the priorities adopted by the websites at the time of their creation, emphasizing the importance of analyzing the several components to ensure a more complete view of the quality of information made available to the population.

The evaluation of each website followed a previously established protocol, covering several aspects of the analysis, in addition to the inclusion of two or more judges for its assessment.^18^ Thus, we aimed to reduce the bias of subjectivity, and enable the replication of this study, increasing the strength of the evidence.

## Conclusion

Most of the websites available to parents or guardians about adenotonsillectomy in children showed a deficit in terms of overall quality based on the assessment of the several aspects of a website (comprehensibility, readability and ethical principles). Therefore, health professionals should include in their clinical routine the indication of websites they consider to be reliable and complete regarding information about adenotonsillectomy, as well as verbal recommendations and the handing out of printed materials in their offices.

## Conflicts of interest

The authors declare no conflicts of interest.
